# Peptidomimetics as Potential Anti-Virulence Drugs Against Resistant Bacterial Pathogens

**DOI:** 10.3389/fmicb.2022.831037

**Published:** 2022-04-18

**Authors:** Osmel Fleitas Martínez, Harry Morales Duque, Octávio Luiz Franco

**Affiliations:** ^1^Programa de Pós-Graduação em Ciências Genômicas e Biotecnologia, Centro de Análises Proteômicas e Bioquímicas, Universidade Católica de Brasília, Brasília, Brazil; ^2^Programa de Pós-Graduação em Biotecnologia, S-Inova Biotech, Universidade Católica Dom Bosco, Campo Grande, Brazil

**Keywords:** peptidomimetics, anti-virulence, biofilms, quorum sensing, secretion systems, resistance

## Abstract

The uncontrollable spread of superbugs calls for new approaches in dealing with microbial-antibiotic resistance. Accordingly, the anti-virulence approach has arisen as an attractive unconventional strategy to face multidrug-resistant pathogens. As an emergent strategy, there is an imperative demand for discovery, design, and development of anti-virulence drugs. In this regard, peptidomimetic compounds could be a valuable source of anti-virulence drugs, since these molecules circumvent several shortcomings of natural peptide-based drugs like proteolytic instability, immunogenicity, toxicity, and low bioavailability. Some emerging evidence points to the feasibility of peptidomimetics to impair pathogen virulence. Consequently, in this review, we shed some light on the potential of peptidomimetics as anti-virulence drugs to overcome antibiotic resistance. Specifically, we address the anti-virulence activity of peptidomimetics against pathogens’ secretion systems, biofilms, and quorum-sensing systems.

## Introduction

The WHO has recognized antimicrobial resistance as a serious global public health threat (World Health Organization, 2021). It is estimated that about 700,000 deaths by antibiotic-resistant infections take place annually, but this quantity could rise to millions in the next few years (Dadgostar, 2019; World Health Organization, 2019). Antibiotic resistance negatively affects medical procedures like surgery, organ transplantation, and chemotherapy (World Health Organization, 2021). The emergence and spread of superbugs have imposed the necessity to look for non-conventional therapy to face this challenge. In this regard, anti-virulence therapy has emerged as an alternative approach to overcome the emergence and spread of antimicrobial-resistant pathogens (Fleitas Martínez et al., 2019a; Krell and Matilla, 2021). Anti-virulence therapy aims to reduce the virulence capacity of pathogens through interference with their virulence factors or virulence-associated processes, trying not to affect the pathogen’s fitness (Fleitas Martínez et al., 2019a; Krell and Matilla, 2021). Such an approach could exert less selective pressure on the selection of resistant pathogens and could make pathogens more susceptible to the host immune system (Liu et al., 2008; Dickey et al., 2017). Since anti-virulence therapy targets virulence factors associated with the pathogen, it should work in a pathogen-specific manner, which should minimize the host-microbiota damage (Dickey et al., 2017).

As a non-traditional therapeutic approach anti-virulence therapy faces translational challenges, so it has been envisioned as an adjunctive therapy more than an alternative therapy to antibiotics (Theuretzbacher and Piddock, 2019; Moir et al., 2021). In this regard, anti-virulence drugs when used in combination with an antibiotic could increase the antibiotics efficacy and diminish their cytotoxic effects (De La Fuente-Núñez et al., 2015; Dickey et al., 2017; Topa et al., 2020). In immunocompromised patients, the pathogens could persist after anti-virulence treatment, due to anti-virulence drugs not kill the pathogens. In this context, the use of combined therapy with antibiotics could be desirable (Hotinger et al., 2021a). Despite being conceived to exert less selective pressure, anti-virulence drugs are not free from the development of resistance by pathogens (Maeda et al., 2012; Maura et al., 2016).

**Figure 1:**
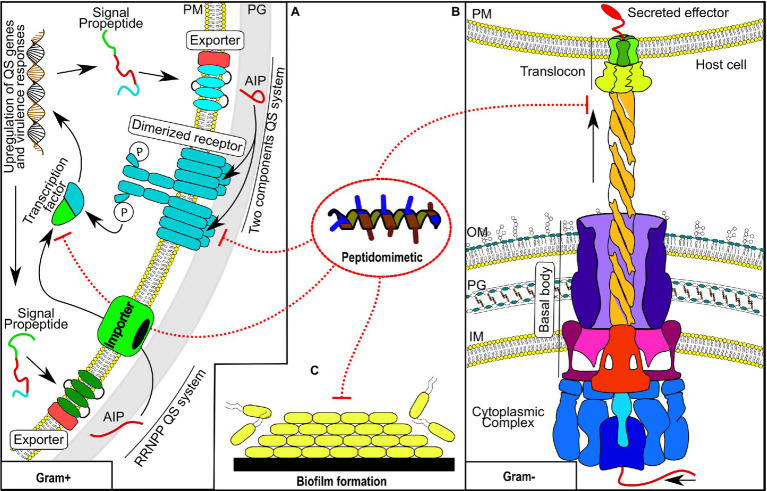
Bacterial virulence-associated processes targeted by peptidomimetics. **(A)** Two quorum-sensing systems from Gram-positive bacteria. **(B)** A representative Gram-negative secretion system. **(C)** Any phase of biofilm formation. QS, quorum sensing; PM, phospholipid membrane; PG, peptidoglycan cell wall; AIP, autoinducing peptides; OM, outer membrane; and IM, inner membrane.

On the other hand, as an emergent strategy, there is an urgent demand for discovery, design, and development of anti-virulence drugs. Natural peptide-based drugs have been identified as promising anti-virulence agents; however, these types of drugs tend to be associated with proteolytic instability, immunogenicity, toxicity, and low bioavailability, which could limit their applicability as anti-virulence agents (Overhage et al., 2008; Kudryashova et al., 2017; Larzabal et al., 2019; Lenci and Trabocchi, 2020; Rezende et al., 2021). In this context, peptidomimetic compounds could be a valuable source of anti-virulence drugs, since they can overcome some of the shortcomings associated with natural peptide-based drugs (De La Fuente-Núñez et al., 2015; Duprez et al., 2015). Although peptidomimetics have been exploited mainly as antimicrobial agents, experimental evidence also supports their potential as anti-virulence drugs (De La Fuente-Núñez et al., 2015; Duprez et al., 2015; Lam et al., 2017; Hershkovits et al., 2019; Cherrak et al., 2021; Lam et al., 2021). Consequently, in this review, we will address some bacterial virulence-associated processes in which peptidomimetics have shown their anti-virulence potential (Figure 1). The activity of peptidomimetics will be described in relation to bacterial secretion systems, bacterial biofilms, and quorum sensing systems, which are intimately linked with bacterial virulence.

Bacterial pathogens use secretion systems to secrete virulence factors that allow them to manipulate and colonize the host environment (Grishin et al., 2021). Experimental evidence supports that interference with bacterial secretion systems could be a feasible strategy to affect the virulence of relevant bacterial pathogens. It has been observed that mutant strains in secretion system components are less virulent than the wild-type strains in animal infection models (Wang et al., 2016; Sanchez-Villamil et al., 2020; Spencer et al., 2021; Taylor et al., 2021). Moreover, the inhibition of secretion systems by drugs also attenuated the pathogen’s virulence *in vivo* (Sheremet et al., 2018; Aburto-Rodríguez et al., 2021; Jia et al., 2021; Zhang et al., 2021; Zigangirova et al., 2021).

Biofilms are a form of bacterial growth that ensures pathogens’ persistence and dissemination (Lebeaux et al., 2014; Batoni et al., 2016; Sahoo et al., 2021). Biofilms are produced by a wide diversity of microbes growing in monomicrobial or polymicrobial communities enclosed in a protective matrix (Preda and Săndulescu, 2019; Luo et al., 2021). Biofilm development is a regulated process, in which second-messengers and quorum-sensing regulatory systems are involved (Mahto et al., 2021). The growth in biofilms confers high resistance against antibiotics and immune system clearance due to the convergence of multiple resistance mechanisms (Taylor et al., 2014; Batoni et al., 2016; Uruén et al., 2021). It is estimated that biofilms are involved in ~80% of all chronic infections and ~65% of all bacterial infections (Preda and Săndulescu, 2019). Biofilm-associated infections are challenged to treat, so several therapeutic strategies are being explored to treat these types of infections (Lebeaux et al., 2014; Fu et al., 2021; Sahoo et al., 2021).

Quorum sensing systems are signaling networks that regulate bacterial behavior and virulence genes expression (LaSarre and Federle, 2013). These communicational systems are intimately linked to bacterial biofilm development since they influence several stages of the biofilm life cycle (Lazar et al., 2021; Mahto et al., 2021). Therefore, interference with quorum-sensing systems is one of the explored strategies to overcome biofilm development by pathogenic bacteria (Brackman and Coenye, 2015; Paluch et al., 2020; Fu et al., 2021). The functionality of quorum-sensing systems resides on basic components that are druggable and tend to be absent in mammals (LaSarre and Federle, 2013; Fleitas Martínez et al., 2019b; Ellermann and Sperandio, 2020). Similarly to the observed in bacterial secretion systems, mutant strains in quorum sensing system components could become avirulent strains in infection models (Askoura et al., 2021; Kumar et al., 2021). Studies focused on quenching quorum sensing systems with inhibitors have demonstrated that targeting these communication systems is a promising strategy to face resistant pathogens (Sully et al., 2014; Daly et al., 2015; Parlet et al., 2019; Salam et al., 2021; Wang et al., 2021).

## Dedicated Secretion Systems

In order to invade, colonize, replicate, and settle successfully in the host, bacterial pathogens use an array of secreted effectors that allow them to manipulate the host environment (Grishin et al., 2021). These secreted effectors are regulated and efficiently delivered by specialized secretion systems (Green and Mecsas, 2016; Grishin et al., 2021). The secretion systems are closely linked with several bacterial pathogenic processes including host invasion and colonization, host immune response evasion, persistence, cytotoxicity and cellular damage, spread, and biofilm formation (Cianciotto and White, 2017; Gorasia et al., 2020; Whelan et al., 2020; Hajra et al., 2021; Rivera-Calzada et al., 2021; Viana et al., 2021; Yu et al., 2021). Consequently, these secretion systems are important for pathogen virulence, making them a target to be explored in anti-virulence therapy (Kimura et al., 2011; Sun et al., 2014; Shaffer et al., 2016; Morgan et al., 2017; Lettl et al., 2020; Cherrak et al., 2021). Currently, nine bacterial secretion systems (I-IX) have been described, from which those majorly targeted by peptidomimetics have been systems III (T3SS) and VI (T6SS) (Lam et al., 2017; Cherrak et al., 2021; Lam et al., 2021).

T3SS is a secretion system associated with flagellum components (Flagellar-T3SS) transport in motile bacteria, but also with virulence effectors transport in pathogenic Gram-negative bacteria through the Non-Flagellar-T3SS system (NF-T3SS) (Hajra et al., 2021). NF-T3SS is a multiprotein complex that transports bacterial virulence effectors directly from the pathogen cytosol into the host cell through a needle-like structure that traverses pathogen and host cell membranes (Hajra et al., 2021; Hotinger et al., 2021b). Structurally, NF-T3SS is composed of five major elements that include the cytoplasmic complex, export apparatus, basal body, needle, and translocon (Hajra et al., 2021; Hotinger et al., 2021b).

Since NF-T3SS is a multiprotein complex, protein–protein interactions could be a key process for the secretion system assembly and functionality (Hajra et al., 2021; Hotinger et al., 2021b). Consequently, interference with the protein–protein interaction network could be a strategy for the development of anti-virulence drugs that target NF-T3SS. In this regard, Stone et al. (2011) showed that a peptidomimetic based on the interaction between proteins CdsN (Type 3 system ATPase) and CdsL in *Chlamydia pneumoniae* inhibited the invasion of HeLa cells by this pathogen in a dose-dependent manner. This peptidomimetic was inspired by one of the binding CdsL domains present in CdsN. Specifically, peptide TRFARA (CdsL binding sequence in CdsN _265–270_) was extended with a CdsN sequence that flanked it. Moreover, it was fused at the N-terminal with a membrane transport sequence (YGRKKRRQRRR), and cysteine residues were also added. These modifications yielded the peptidomimetic YGRKKRRQRRRCVVLMMDSVTRFARALC. The membrane transport sequence inclusion in the peptidomimetic facilitated its penetration of *C. pneumoniae* infectious elementary bodies (Stone et al., 2011).

Synthetic coiled-coil peptides also interfered with the assembly and functionality of NF-T3SS in enteropathogenic *Escherichia coli* (EPEC), enterohemorrhagic *E. coli* O157:H7 (EHEC), and the murine pathogen *Citrobacter rodentium* (Larzabal et al., 2010, 2013). These peptides appear to act by disrupting the oligomerization of the NF-T3SS system EspA protein by interacting with the C-terminal coiled-coil domain of EspA (Larzabal et al., 2019). Coiled-coil peptides protected mice from *C. rodentium* infection, avoiding intestinal damage (Larzabal et al., 2013). Although they are not peptidomimetics, the promising selective inhibitory activity against NF-T3SS exerted by coiled-coil peptides, as well as their tolerability and safety in animal models, make them a valuable scaffold for the development and design of novel peptidomimetic with enhanced anti-virulence activity.

**Figure 2:**
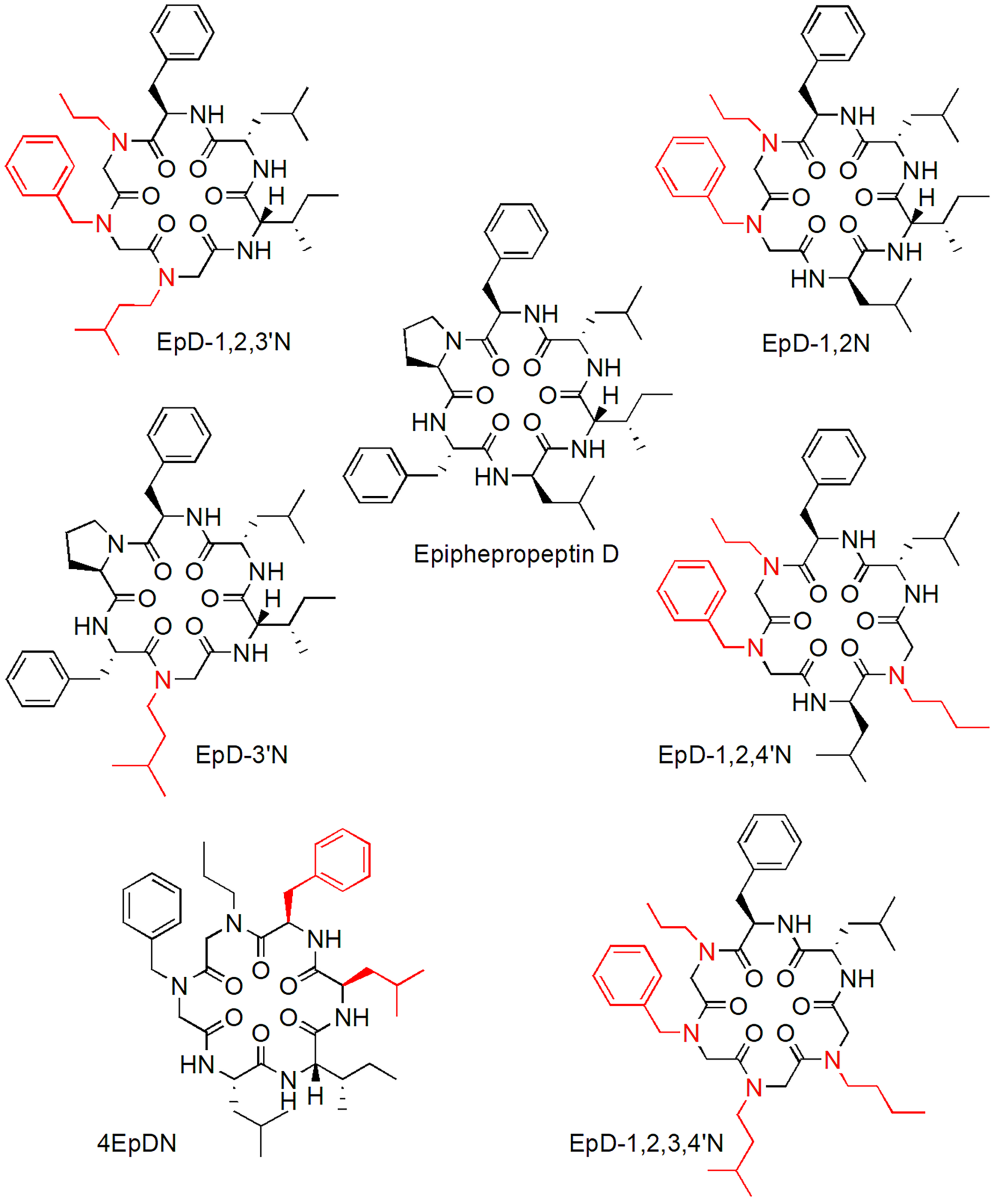
Cyclic peptomers with inhibitory activity against bacterial NF-T3SS secretory system.

Cyclic peptomers (cyclic peptide-peptoid hybrid molecules) are a new NF-T3SS peptidomimetic inhibitors class derived from epiphepropeptin D (EpD), a stereoisomer of the cyclic natural hexapeptide phepropeptin D. They selectively inhibit NF-T3SS activity without affecting bacterial growth or flagellar motility and without exerting considerable toxic effects on mammalian cells (Figure 2; Lam et al., 2017). Cyclic peptomers with single peptoid substitutions on the cyclic peptide backbone significantly inhibited the *Yersinia pseudotuberculosis* NF-T3SS system (Lam et al., 2017). However, the introduction of multiple peptoid substitutions at positions 1, 2, 3, and 4 on the peptide backbone yielded new peptomers with enhanced *Y. pseudotuberculosis* NF-T3SS system dose-dependent inhibition (Figure 2; Lam et al., 2017). Cyclic peptomers also inhibited the *Pseudomonas aeruginosa* NF-T3SS system in a dose-dependent fashion. Specifically, the peptomers EpD-3’N, EpD-1,2 N, EpD-1,2,3’N, EpD-1,2,4’N, and EpD-1,2,3´,4’N were the most active against *Y. pseudotuberculosis* and *P. aeruginosa* NF-T3SS systems (Figure 2; Lam et al., 2017). Moreover, cyclic peptomers inhibited the activity of *Y. pseudotuberculosis* effector proteins in HeLa cells as well as their translocation into CHO-K1 cells *via* NF-T3SS. Particularly, EpD-3’N and EpD-1,2,4’N exerted inhibitory activity of *Y. pseudotuberculosis* effector proteins in a range of concentrations of 3.75–60 and 1.875–60 μM, respectively, whereas EpD-1,2,3’N (1.875–15 μM), EpD-1,2,3´,4’N (1.875–15 μM), EpD-4’N (1.875–30 μM), and EpD-1,3’N (1.875–7.5 μM) exerted some inhibitory activity at a narrower concentration range. Nevertheless, some of these peptomers at concentrations greater than or equal to 15 μM appear to produce undesirable effects on the host actin cytoskeleton (Lam et al., 2017).

A promising cyclic peptomer (4EpDN) selectively inhibited NF-T3SS systems belonging to different T3SS families and different pathogens, such as *Y. pseudotuberculosis*, *Yersinia enterocolitica*, *P. aeruginosa*, *Salmonella enterica* serovar Thyphimurium, and *Chlamydia trachomatis*, without affecting bacterial growth (Lam et al., 2021). 4EpDN was derived from the previously reported peptomer EpD-1,2 N (Figure 2) *via* stereochemistry scan. Specifically, 4EpDN and EpD-1,2 N differ at positions 3 and 5 by D, L-leucine stereoisomers (Figure 2). Immunofluorescence microscopy analysis of 4EpDN-treated *Y. pseudotuberculosis* showed a reduction in the number of YscF punctates on bacteria (YscF is a T3SS needle subunit in *Yersinia*), suggesting that 4EpDN probably operates by interfering with the assembly or stability of T3SS needles, which impair the secretion of virulence factors. A relevant fact was that 4EpDN inhibited chlamydial infection of HeLa cells (Lam et al., 2021). In general, the experimental evidence provided by these studies suggests that cyclic peptomers are promising anti-virulence agents that act as broad-spectrum NF-T3SS inhibitors.

The type VI secretion system (T6SS) could also be targeted by peptidomimetics (Cherrak et al., 2021). T6SS has been associated with virulence in several bacterial pathogens, including some ESKAPE group members (*Enterococcus faecium*, *Staphylococcus aureus*, *Klebsiella pneumoniae*, *Acinetobacter baumannii*, *P. aeruginosa*, and *Enterobacter cloacae*), which are a serious threat for the human health due to the development of high resistance levels against a broad spectrum of antibiotics (Boyer et al., 2009; Wang et al., 2015; Kim et al., 2017; Zhou et al., 2020). Like to NF-T3SS, T6SS is a multiprotein complex for whose assembly the protein–protein interactions could be essential (Cherrak et al., 2021). Therefore, the T6SS protein–protein interaction network could be exploited for the development of anti-virulence drugs. A rationally designed cycled peptide has recently been reported that interferes with the interaction between TssK (trimer) and TssG, which impaired the T6SS baseplate biogenesis in the enteroaggregative *E. coli* (EAEC) (Cherrak et al., 2021). TssK and TssG interact *via* two-foot regions (foot 1 and 2) in TssG and by an N-terminal β sandwich shoulder domain (NTD) in TssK. The foot regions on TssG have a triangular fold and contain a conserved sequence pattern, LGXXXX^1^LGXXXX^2^LG (LG repeats), which harbor conserved hydrophobic amino acid residues (L or M) in certain positions, followed by small or basic residues (G, S, R, and K). The XXXX^1^ sequence is variable in composition and length, whereas the XXXX^2^ sequence is less variable. The TssK NTD domain has a C3-symmetric shape and harbors hydrophobic cavities involved in the interactions with the LG repeats motif of TssG. Hydrophobic interactions are essential for TssK-TssG interaction stability. Based on this structural information, a biomimetic cyclic peptide (BCP) (SRPVMG-SRPVMG-SRPVMG) was designed, mimicking the interaction interface between TssG foot domains and the TssK NTD domain (Cherrak et al., 2021). BCP was designed from TssG foot 1 and cycled *via* head-to-tail cyclization. The BCP cyclic structure allowed it to fit better with the C3-symmetric shape of the TssK NTD domain. *In vitro* assays showed that BCP binds to TssK and interferes with TssKFGE complex formation (baseplate wedge complex). Moreover, in permeabilized EAEC bacteria treated with BCP, toxic effects were not observed, and there was a reduction in the number of TssK-sfGFP containing foci. Interestingly, it was observed that pathogenic bacteria containing T6SS systems show high conservation of the TssK motif targeted by BCP. The TssK-TssG interacting interface is also conserved. Such facts suggest that BCP could exert a broad-spectrum inhibitory activity or could be a scaffold for anti-T6SS broad-spectrum drug development (Cherrak et al., 2021). Despite its promising activity, BCP needs improvement in issues associated with cell membrane diffusion, affinity, and stability.

Bacterial secretion systems have been recognized as an attractive target to develop anti-virulence drugs. A diversity of compounds that inhibit bacterial secretion systems attenuated pathogen virulence in animal models (Hudson et al., 2007; Larzabal et al., 2013; Sawa et al., 2014; Berube et al., 2017; Sheremet et al., 2018; Feng et al., 2019; Ngo et al., 2019). It suggests that these systems are a feasible target and that compounds that target them could act as anti-virulence drugs. Most of the experimental evidence about peptidomimetic inhibitory activities against bacterial secretion systems reside on *in vitro* assays. Therefore, the design and development of studies in animal models are imperative to have convincing evidence of their anti-virulence activity.

## Peptide-Based Quorum Sensing Modulators in Bacteria

Autoinducer peptides (AIPs) are components of a signaling system used by some pathogenic microorganisms, mainly Gram-positive bacteria, to invade their hosts (LaSarre and Federle, 2013). AIPs are short peptides whose amino acid content varies between seven to twelve residues. In some cases, their cyclized C-terminal is a structural requirement for activity (LaSarre and Federle, 2013; Khan et al., 2015). Peptidomimetics have been studied with regard to peptide-based quorum sensing (QS) with two principal objectives. Peptidomimetics were used to identify structure–activity relationships (SARs) of natural AIPs and to develop new protease-resistant compounds. Peptide-based QS comprises two system mechanisms typical of Gram-positive bacteria (Figure 1), whose final function is to modulate virulence, communication, and/or antibiotic resistance through the expression or repression of proteins (McBrayer et al., 2020). The first one, which has been studied extensively (Li and Rebuffat, 2020), is called the two-component pathway system (Wu et al., 2020). Typically, such a system comprise two transmembrane proteins responsible for AIP secretion and later interaction for activity, respectively (Khan et al., 2015; Wu et al., 2020). It has a transcription factor responsible for the QS system expression, which is activated after phosphorylation by a dimerized receptor (Khan et al., 2015; McBrayer et al., 2020). Some categories of this system are described as the accessory gene regulator (Agr system) from *S. aureus* (Jenul and Horswill, 2019), the competence operon (Com system) from *Streptococcus pneumoniae* (Chandler and Morrison, 1987), and the system regulator (Fsr system) from *Enterococcus faecalis* (Qin et al., 2000). The second system involves the pathway of response regulators (RRNPP) (Perez-Pascual et al., 2016). The identified RRNPP are (**P**lcR) and (**N**prR) from *Bacillus cereus* (Uehara et al., 1974; Agaisse et al., 1999), (**R**ap) from *Bacillus subtilis* (Jiang et al., 2000), (**P**rgX) from *E. faecalis* (Kozlowicz et al., 2006), and (**R**gg) from *Streptococcus* (Ibrahim et al., 2007).

The pathway of two components typically includes some membranal proteins (proteases and signal transduction) system, which allows maturation of macrocyclic peptides and extracellular signalization, respectively (Wu et al., 2020). For *Staphylococci* spp., the AgrA response regulator allows P2 and P3 promotors to be acted on, which upregulate the RNAII (*agr* operon) and RNAIII effector molecules, respectively (Jenul and Horswill, 2019). RNAIII is responsible for virulence compound expression (Morfeldt et al., 1995). The *agr* operon transcription translates AgrA-D proteins responsible for autoinducing and intra- or interspecific communication (Novick et al., 1995). The AgrD protein is the immature AIP (Ji et al., 1995), which should be cleaved and secreted by Sps and AgrB membranal proteins, respectively (Thoendel and Horswill, 2009, 2013). When the mature AIP is exported, it acts on the AgrC receptor, initializing a phosphorylation signal from the AgrC receptor to the AgrA regulator (Novick et al., 1995; Lina et al., 1998). The phosphorylated AgrA can upregulate P2 and P3 promotors to close the cycle (Koenig et al., 2004).

There are four (I-IV) types of AIP in *Staphylococcus* species (Jenul and Horswill, 2019). For *S. aureus*, each AIP is an activator of its cognate receptor (AgrC I-IV) but an inactivator for other receptors (Wang and Muir, 2016). Only AIP IV is an agonist on both AgrC I and IV receptors. However, for *S. epidermidis*, AIP I is an AgrC I activator, but an AgrC II-III antagonist, while AIPs II-III are AgrC II-III activators, but AgrC I antagonists (West et al., 2021). Studies have shown that AIPs’ agonism or antagonism activity could be correlated to twist the linker interdomains (between sensor and HK) of the ArgC receptor (Wang et al., 2017b). Activation or inhibition performed by ArgC receptors could be governed by the opposite rotation of such a linker promoted by AIP binding (Wang and Muir, 2016). Some AIP features are responsible for peptide/argC interaction. In principle, natural AIPs should have their C-terminal extremity cyclized (Khan et al., 2015). It is presumed that macrocyclic structure favors circular and planar disposition of side-chains in AIP amino acids for ArgC interaction (Horswill and Gordon, 2020). Unfortunately, for synthetic AIPs, macrocycle synthesis seems to be an obstacle to clinical development (Gordon, 2020). However, some linear peptidomimetics (peptide and peptoid mixture) were shown to be effective competitive antagonists of the AgrC receptor (Karathanasi et al., 2018). In that case, linear structures decrease synthesis steps, costs of production, and they may also become more stable. Interestingly, the peptide counterparts (A3, D3, and G3) of prominent peptidomimetics showed no anti-virulence activity (Karathanasi et al., 2018). This could be explained by the structural conformation of the peptides in solution (Tal-Gan et al., 2016). While natural peptides could adopt β-sheet or α-helical structures, these peptoids would interrupt secondary structures, facilitating specific disposition of the side-chain length necessary for receptor interaction. Nevertheless, this is not a general rule. For the *S. pneumoniae* Com system, AIPs (named CSP1 and CSP2) showed some degree of helicity necessary for AIP/cognate receptor interaction (Yang et al., 2017). The fundamental activity may occur due to a hydrophobic patch induced by secondary peptide structure (McBrayer et al., 2020). An example of this phenomenon is CSP2-d10, whose D-amino acid did not interrupt such a hydrophobic patch and therefore maintained the agonistic activity (Yang et al., 2017).

For *S. epidermidis*, some amino acids in the endocyclic segment (9, 10, 11, and 12) are important for AIP II-III/ArgC interactions, while exocyclic amino acids seem to be efficient for AIP switch activity (agonism/antagonism) (West et al., 2021). For AIP I/ArgC interaction, amino acids positioned at 3, 5, 6, 7, and 8 were seen to be important for AIP I/ArgC interaction, and the third residue was responsible for switch activity (Yang et al., 2016). Peptidomimetics involving ArgC interactions are shown in Table 1. For *S. aureus*, for example, a D-enantiomer scan in AIP III revealed that (Ile1 and Asp2) substitutions were potent inhibitors of AgrC I-IV receptors, while other substitutions showed a preference for some receptors or low activity on them (Tal-Gan et al., 2013). Interestingly, three acetylated derivatives of the same AIP showed more potent antagonism on the four-receptor type than D-enantiomer counterparts (Tal-Gan et al., 2013). AIP acetylation on the N-terminal had previously been identified. This modification, substituting extracyclic amino acids in AIP II, showed to be a shift that would turn agonist AIPs into antagonists in the AgrC system (Lyon et al., 2002). It is suggested that the exocyclic tail from AIPs should be extended far from the macrocycle for agonist activity, while in antagonist activity, the tail should be folded over the macrocycle (Horswill and Gordon, 2020).

**Table 1:** Peptidomimetics that affect peptide-based quorum sensing system.

Name	Sequence^*^	Agonism	Antagonism	References
*Agr system*				
AIP III	IN(CDFLL)	AgrC III		Tal-Gan et al., 2013
AIP III _D_I1	_D_IN(CDFLL)		AgrC I-IV	
AIP III _D_N2	I_D_N(CDFLL)		AgrC I-IV	
AIP III _D_C3	IN(_D_CDFLL)		Low activity	
AIP III _D_D4	IN(C_D_DFLL)		AgrC I, II, IV	
AIP III _D_F5	IN(CDDFLL)		Low activity	
AIP III _D_L6	IN(CDF_D_LL)	AgrC II	Low activity	
AIP III _D_L7	IN(CDFL_D_L)	AgrC III	AgrC I, II, IV	
tAIP III _D_2A	Ac-(CAFLL)		AgrC I-IV	
tAIP III D2A/F3Y	Ac-(CAYLL)		AgrC I-IV	
tAIP IIID2A/F3W	Ac-(CAWLL)		AgrC I-IV	
AIP II	GVNA(CSSLF)	AgrC II		Tal-Gan et al., 2016; Vasquez et al., 2017
tAIP II	Ac-(CSSLF)		AgrC I-IV	
n5FF	Ac-(C_5Me_FF)		AgrC I-IV	
n6FF	Ac-(C_6Me_FF)		AgrC I-IV	
n7FF	Ac-(C_7Me_FF)		AgrC I-IV	
Peptide A3	WFAKMWK		Inactive	Karathanasi et al., 2018
A3	Analogous peptoid		AgrC I	
Peptide D3	WFKKMWK		Inactive	
D3	Analogous peptoid		AgrC I	
Peptide G3	WFGKMWK		Inactive	
G3	Analogous peptoid		AgrC I	
AIP II	GVNA(CSSLF)	AgrC II		Vasquez and Blackwell, 2019
tAIP II	Ac-(CSSLF)		AgrC I-IV	
n7OFF	Ac-(C_7Me2O_FF)		Low activity	
Bnc3	Phe-Ac-(C_7Me2Ocp_A_3f_F)		AgrC I-IV	
Pentyl-n7OCpa(3fF)			AgrC I-IV	
Bn-n7OCpa(3C1F)			AgrC I-IV	
Bn-n7OCpa(3fF)-am			AgrC III	
Bn-n7OCpa(3fF)-es			AgrC III	
Decanoyl-ornithyl-ornithyl-dodecanoyl-ornithyl-amide	C_10_OOc_12_O (Lipopeptide)		Agr-MRSA	Hershkovits et al., 2019
AIP II	NASKYNP(CSNYL)	AgrC II	AgrC I	West et al., 2021
AIP II _D_K4	NAS_D_KYNP(CSNYL)	Low activity		
AIP II _D_S9	NASKYNP(C_D_SNYL)	Inactive		
AIP II _D_N10	NASKYNP(CS_D_NYL)	Inactive		
AIP III	NAAKYNP(CASYL)	AgrC III	AgrC I	
AIP III _D_K4	NAA_D_KYNP(CASYL)		AgrC III	
AIP III _D_A9	NAAKYNP(C_D_ASYL)	Inactive		
AIP II _D_S10	NAAKYNP(CA_D_SYL)	AgrC III		
*Com system*				
CSP1	EMRLSKFFRDFILQRKK	ComD1		Yang et al., 2017
		Low activity on ComD2		
CSP1-_D_S5	EMRL_D_SKFFRDFILQRKK	ComD1		
CSP1-_D_Q14	EMRLSKFFRDFIL_D_QRKK	ComD1		
CSP1-_D_R15	EMRLSKFFRDFILQ_D_RKK	ComD1		
CSP1-_D_K16	EMRLSKFFRDFILQR_D_KK	ComD1		
CSP1-_D_K17	EMRLSKFFRDFILQRK_D_K	ComD1		
CSP2	EMRISRIILDFLFLRKK	ComD2		
		Low activity on ComD1		
CSP2-_D_F13	EMRISRIILDFL_D_FLRKK	ComD2		
CSP2-_D_L14	EMRISRIILDFLF_D_LRKK	ComD2		
CSP2-_D_R15	EMRISRIILDFLFL_D_RKK	ComD2		
CSP2-_D_K16	EMRISRIILDFLFLR_D_KK	ComD2		
CSP2-_D_K17	EMRISRIILDFLFLRK_D_K	ComD2		
CSP2-E1A _D_D10	AMRISRIIL_D_DFLFLRKK		ComD2	
CSP2-E1A I4N_vaD_10L14Q	AMR**Nva**SRIIL_D_FLFQRKK		ComD1 and 2	Koirala and Tal-Gan, 2020
CSP1-E1A-cyc(Dap6E10)	AMRLS(**Dap**FFRE)FILQRKK		ComD1 and 2	Yang et al., 2020
CSP1-E1A-desK16 K17cyc(Dap6E10)	AMRLS(**Dap**FFRE)FILQR		ComD1 and 2	
18-CSP	SGSLSTFFRLFNRSFTQA	ComD		Bikash et al., 2018
18-CSP(_D_L4)	SGS_D_LSTFFRLFNRSFTQA	ComD		
18-CSP(_D_S1)	DSGSLSTFFRLFNRSFTQA	ComD		
P3	18-CSP-des-S1G2-*N*-Me-S3	ComD		Bikash and Tal-Gan, 2019
*Fsr system*				
GBAP	QN(SPNIFGQWM)	FsrC		McBrayer et al., 2017
GBAP-_D_Q1	DQN(SPNIFGQWM)	FsrC		
GBAP-_D_N2	Q_D_N(SPNIFGQWM)	FsrC		
Ac-GBAP	Ac-QN(SPNIFGQWM)	FsrC		
Ac-GBAP-DesQ1	Ac-N(SPNIFGQWM)	FsrC		
Ac-GBAP-desQ1N2	Ac-(SPNIFGQWM)	FsrC		
GBAP-N5[YBzl]M11A	QN(SP-YBzl-IFGQWA)		FsrC	McBrayer et al., 2018
Ac-GBAP-Des (Q1N2)[*N*-MeF7]	Ac-(SPNI[*N*MeF7]GQWM)	FsrC		McBrayer et al., 2019
ZBzl-YAA5911	Z-QN(SP[YBzl]IFGQWM)		FsrC	Nakayama et al., 2013
*RRNPP pathway system*				
PapR_7_	ADLPFEF	PlcR		Yehuda et al., 2018
PapR7-_D_A1	DADLPFEF	PlcR		
PapR7-_D_E6	ADLPF_D_EF		PlcR	
PapR7-_D_F7	ADLPFE_D_F		PlcR	

t, truncated or lacking an N-terminal exocyclic tail; Agr, accessory gene regulator; Com, competence operon; Dap, 2,3-diaminopropionic acid; Fsr, system regulator; Nva, norvaline; and RRNPP, pathway of response regulators

* Macrocyclic structures are indicated in parentheses when necessary.

It is not surprising that some modified AIPs without extracyclic amino acids show antagonist activity with similar potency to those natural agonists (Tal-Gan et al., 2016). A study of 63 peptidomimetics was developed. Among them, three acetylated homologues of AIP II, with two hydrophilic intracyclic amino acids substituted by methylene (*n* = 5–8), showed antagonist activity on AgrC I-IV (Vasquez et al., 2017). A battery of synthetic analogues was developed from a peptidomimetic (n7OFF) designed by the authors (Table 1). Among them, five peptidomimetics showed prominent anti-virulence activity, of which Bnc3 was the most potent (Vasquez and Blackwell, 2019). These last investigations involved chemical modifications into the AIPs. So far, antagonistic activity of peptidomimetics has been developed from D-enantiomers, acetylated peptoids, linearized AIPs, or those with chemical modifications. Also, peptidomimetics with additional modification showed an effect on *S. aureus* (Table 1). The lipopeptide-like AIP, decanoyl-ornithyl-ornithyl-dodecanoyl-ornithyl-amide (C_10_OOc_12_O), had anti-virulence activity on the Agr-MRSA system (Hershkovits et al., 2019). A current study made on other *Staphylococci* spp. shows that a modified AIP makes the difference in AIP/receptor activity. D-enantiomers of AIP III, from *S. epidermidis*, were tested on AgrC system. Here, the Ser10 epimer held its agonist activity, but when the substitution was on the Lys4 epimer, the peptide showed a strong antagonist activity (West et al., 2021). Other substitutions by D-enantiomers in AIPs II and III reported in this work did not show positive results.

SAR studies with peptidomimetics in Com and Fsr systems appear less in the literature. These systems are composed of homologous proteins of the *agr* system (Gray et al., 2013; Li and Rebuffat, 2020). A histidine kinase (ComD and FsrC, respectively) is responsible for AIP interaction and the signaling for transcription regulation (Gray et al., 2013). In a competence-inducing QS system, cyclic structure in AIPs seems not to be a requirement (LaSarre and Federle, 2013). Like AIP in the *agr* system, the competence stimulating peptide (CSP) is responsible for targeting ComD receptors (Novick and Geisinger, 2008). In such a system, there are two CSP types (CSP1 and CSP2) for their respective cognate targets (Pozzi et al., 1996). A systematic D-enantiomer scan was done using CSP1 and CSP2 on receptors from *S. pneumoniae* (Table 1). Among the variants, some CSP1 or CSP2 analogs enhanced the agonistic activity, but only a CSP2 variant (CSP2-E1ADD10) had potent antagonistic activity on ComD2 (Yang et al., 2017). Curiously, unlike AIPs in *Staphylococci* spp., each CSP type is not an antagonist of its noncognate receptor. Subsequent modifications led to the most potent antagonist targeting both ComD1 and ComD2 receptors (Koirala and Tal-Gan, 2020). This and another 13 analogs, with norvaline and D-enantiomer substitutions in the fourth and tenth positions, respectively, showed antagonistic activity on the ComD2 receptor (Koirala and Tal-Gan, 2020). The last of them to be developed, CSP1-E1A-cyc (Dap6E10), increased its resistance to degradation, and was nontoxic *in vivo* (Table 1). CSP1-E1A-cyc and another cyclized analog that has the Lys4 substituted with 2,3-diaminopropionic acid showed ComD1 and ComD2 antagonism (Yang et al., 2020). Furthermore, D-enantiomers or *N*-methyl analogs hold agonistic activity on *S. mutans* ComD receptors (Table 1). Importantly, the 18-CSP(_D_L4) epimer showed enhanced activity in comparison with its natural counterpart (Bikash et al., 2018). Moreover, none of the *N*-methyl peptidomimetics surpassed the original analog’s activity (Bikash and Tal-Gan, 2019). ComD inhibition activity by these peptidomimetics was not detected (Bikash and Tal-Gan, 2019).

Some SAR studies were made with the Fsr system from *E. faecalis* (Table 1). In this system, the AIP named gelatinase biosynthesis-activating pheromone (GBAP) is responsible for FsrC receptors’ interaction (Qin et al., 2000). Like AIPs in *Staphylococci* spp., GBAPs are cyclized on their carboxyl extremity (LaSarre and Federle, 2013). From three libraries made with modified GBAP (D-enantiomers and alanine scanned and acetylated), some analogs were discovered with equivalent or more potent agonistic activity, but others with low antagonistic activity (McBrayer et al., 2017). To investigate the best analog (Ac-GBAP-desQ1N2), an *N*-methyl scan was performed. Only the Ac-GBAP-Des(Q1N2)[*N*-MeF7] analog showed similar agonistic activity (McBrayer et al., 2019). Fortunately, for this system, some antagonistic candidates were developed (Table 1). ZBzl-YAA5911, an analog with N-terminal benzyloxycarbonylated (Z) and the Tyr5 added to the benzyl (Bzl) group, was the most potent both *in vitro* and *in vivo* (Nakayama et al., 2013).

The RRNPP pathway system has a mechanism like the two-component pathway system. In this system, AIP is translated, exported, and internalized again for target interaction (Perez-Pascual et al., 2016). It is also involved in intra- and interspecific communications (Verdugo-Fuentes et al., 2020). Unlike the pathway of the two-component system, previously mentioned, this system imports the respective AIP to target intracellular factors (Perez-Pascual et al., 2016; McBrayer et al., 2020). Few SAR studies have been done using this system. Due to the intracellular target localization, research into AIPs from the RRNPP pathway may be more difficult. An additional challenge could be the design of peptides to move across the membrane (Wu et al., 2020). Several compounds, which target diverse points in the AIP system, have been investigated to achieve QS modulation in Gram-positive bacteria (Fleitas Martínez et al., 2019b). For example, to inhibit the AIP/cognate receptor interaction of the Rgg system, some drug-like molecules have been showed to be successful (Aggarwal et al., 2015). However, these molecules do not have a peptide nature. Among the PlcR, NprR, Rap, PrgX, and Rgg systems identified until now (Perez-Pascual et al., 2016; McBrayer et al., 2020), only PlcR was approached from this perspective (Table 1). The AIP (named PapR from this system) is responsible for the tetratricopeptide repeat (TPR)-type regulatory domain of PlcR interaction (Bouillaut et al., 2008). For *Bacillus cereus*, a D-enantiomer scan of PapR_7_ showed two analogs with potent antagonistic activity (Yehuda et al., 2018). Another analog displayed more potent agonistic activity than its original counterpart. Overall, for the RRNPP pathway, it is necessary to increase studies to reveal molecular mechanisms for AIPs/cognate receptor interaction. Importantly, attention is called to develop peptidomimetics for anti-virulence activity on pathogenic bacteria.

Several peptidomimetics have been used as pharmacological tools to test AIPs-QS system interactions (Table 1). Most of them showed promising *in vitro* activities. Unfortunately, few works identified the efficacy of these peptidomimetics *in vivo*. Three peptidomimetics that exerted anti-virulence activities were reported, curiously, belonging to Fsr and Com systems. ZBzl-YAA5911 protected against the retinal damage in a rabbit endophthalmitis model (Nakayama et al., 2013), while GBAP-N5[YBzl]M11A, another GBAP analog, inhibited the formation of biofilm by wild-type *E. faecalis* (McBrayer et al., 2018). CSP1-E1Acyc(Dap6E10), an AIP from the Com system, attenuated the mortality caused by *S. pneumoniae* in an acute pneumonia mouse model (Yang et al., 2020). The studies suggest that peptidomimetics that exert a quorum-sensing inhibitory activity can confer protection against pathogens. However, more research must be conducted on this topic.

## Biofilm Disruption by Peptidomimetics

Bacterial biofilms are a predominant type of bacterial growth, where a defensive extracellular polymeric matrix composed mainly of proteins, DNA, RNA, polysaccharides, water, and ions protect the bacteria (Sahoo et al., 2021). The persistence, dissemination, and virulence of bacterial pathogens are facilitated by growing in biofilms (Sahoo et al., 2021). The high antibiotic resistance in biofilms is associated with the convergence of several intrinsic, acquired and adaptive resistance mechanisms (Taylor et al., 2014; Uruén et al., 2021). These mechanisms comprise reduced penetration and sequestering of antimicrobials by the extracellular matrix, high levels of degradative enzymes, horizontal resistance gene transfer, and metabolically heterogeneous bacterial population including persisters (Taylor et al., 2014; Bellich et al., 2018). To develop anti-virulence therapies, biofilms are attractive targets, due to their involvement in virulence and antibiotic resistance.

The treatment of biofilm-associated infections with conventional antimicrobials is very challenging since biofilms are highly heterogeneous entities (structurally, temporarily, and spatially), where multiple resistance mechanisms converge (Taylor et al., 2014; Batoni et al., 2016). Consequently, alternative therapies are being explored against biofilms, and anti-virulence therapy is one of them (Fleitas Martínez et al., 2019a; Xu et al., 2021). Anti-virulence therapy aims to impair biofilm formation and development, trying to exert a marginal effect on pathogen viability (Fleitas Martínez et al., 2019a). In this regard, some peptidomimetics have shown anti-biofilm activity at sub-inhibitory concentrations [in reference to minimum inhibitory concentration (MIC)] (De La Fuente-Núñez et al., 2015).

**Figure 3:**
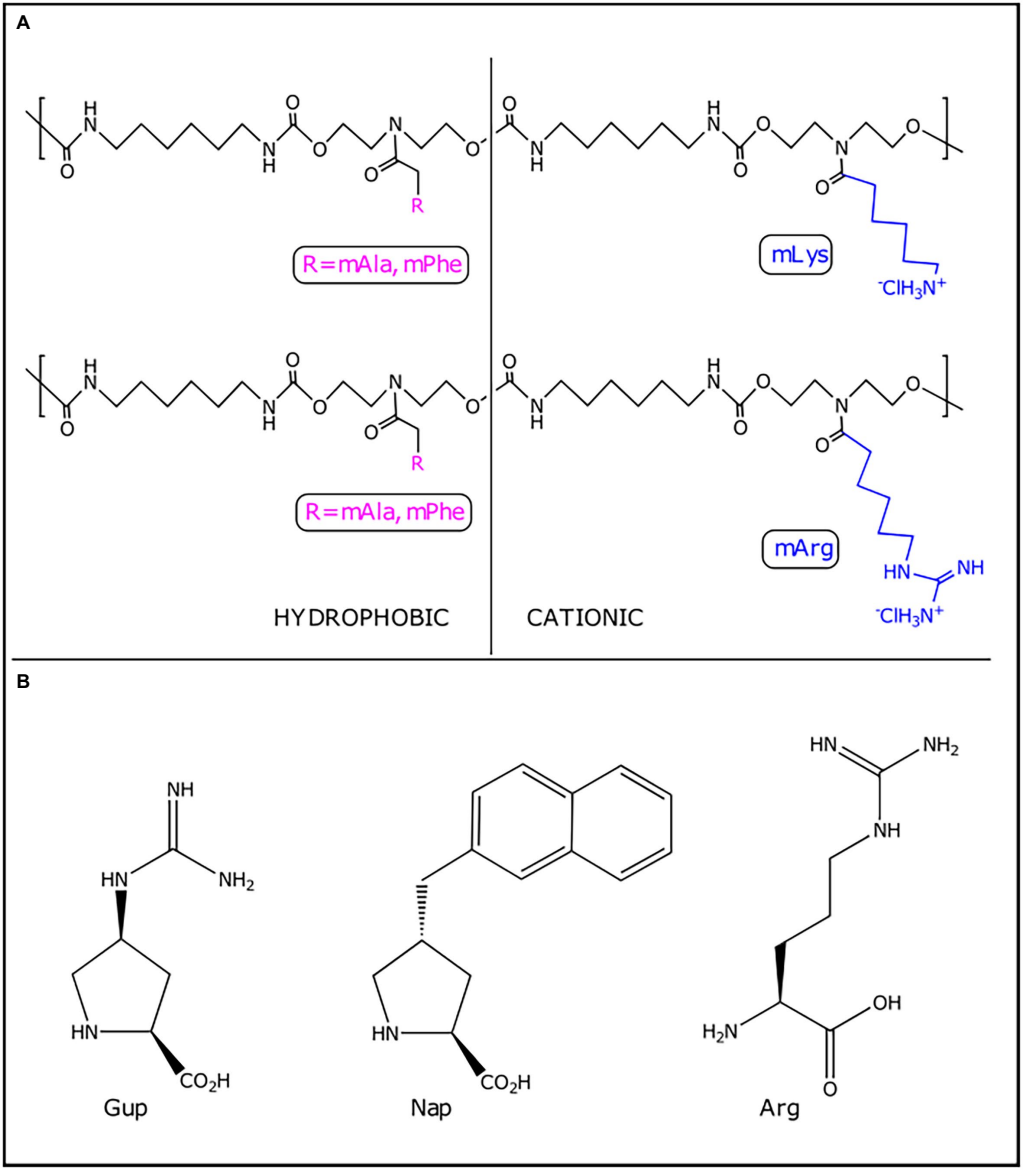
Peptidomimetics with anti-biofilm activity. **(A)** Synthetic polyurethanes. **(B)** Key residues in functionalized proline-rich peptides. Gup, guanidinoproline; Arg, arginine; and Nap, naphthylproline.

The first step to biofilm formation is the reversible deposition and attachment of planktonic bacterial cells on surfaces. The blockage of this initial step could impair biofilm establishment. Four synthetic polyurethanes (mLys/mPhe, mLys/mAla, mArg/mAla, and mArg/mPhe) that mimic anti-biofilm peptides were reported (Table 2; Vishwakarma et al., 2021). Each synthetic polyurethane consisted of charged and hydrophobic pendant groups with a charge-to-hydrophobicity ratio of 60/40. The charged pendant groups mLys and mArg structurally mimic lysine and arginine amino acids, respectively, whereas hydrophobic pendant groups mPhe and mAla structurally mimic phenylalanine and alanine amino acids, respectively (Figure 3; Vishwakarma et al., 2021). Polyurethane antimicrobial activity appears to be affected in enriched mediums like Lysogeny Broth (LB) and Muller Hinton Broth (MH) due to complexation with protein present at media and poor solubility at concentration higher than 250 μg.ml^−1^. At 0.25 x *Minimum Biofilm Inhibitory Concentration* (MBIC), synthetic polyurethanes notoriously affected the attachment of *P. aeruginosa* PAO1 to a glass surface when compared with the cationic cyclic AMP polymyxin B (~0.5× MBIC) and ciprofloxacin (0.5× MBIC). The most active synthetic polyurethane was mLys/mPhe. Zeta-potential measurements of synthetic polyurethane-treated cells suggest that these peptidomimetics modify the superficial charge of *P. aeruginosa* PAO1. The changes in bacterial surface charge induced by the synthetic polyurethanes may constitute one of the mechanisms by which they interfere with the bacterial adherence to the abiotic surfaces since electrostatic interactions between bacteria and surfaces are important to the initial attachment (Tuson and Weibel, 2013). Experimental evidence based on membrane permeability assay using N-phenyl-1-naphthylamine (NPN) points to non-membranolytic interactions between synthetic polyurethanes and bacteria, because polyurethanes do not provoke bacterial membrane permeabilization (Vishwakarma et al., 2021). Consequently, the authors suggested that, at low concentrations, synthetic polyurethanes inhibit biofilm formation by interacting with the bacterial surface in such a way that bacterial attachment is impaired without killing the bacteria (Vishwakarma et al., 2021). An interesting observation was that the attached *P. aeruginosa* PAO1 cells, when treated with ciprofloxacin, showed a filamentous appearance, which has been associated with antibiotic resistance development (Bos et al., 2015). However, the synthetic polyurethane-treated cells do not show this filamentous phenotype. Further experimental evidence showed that mLys/mAla and mLys/mPhe stimulated twitching motility, whereas mArg/mAla and mArg/mPhe noticeably reduced swarming motility. Twitching motility stimulation and swarming motility inhibition have been identified as mechanisms through which AMPs exert their anti-biofilm activity (Overhage et al., 2008; De La Fuente-Núñez et al., 2012). Synthetic polyurethanes appear to interfere with pathogen biofilm development through modulation of bacteria-surface interactions, and their anti-biofilm activity is exerted without toxicity toward mammalian cells, which made them attractive anti-virulence agents (Table 2).

**Table 2:** Peptidomimetics with anti-biofilm activity at sub-inhibitory concentrations.

Peptidomimetics	MIC (μg.ml^−1^)	MBIC (μg.ml^−1^)	Target organism	Mechanism	HC_50_^c^ (μg.ml^−1^)	References
mLys/mPhe	8	8	*P. aeruginosa*	Changes in bacterial surface properties that prevent the attachment Stimulating twitching motility	260.5	Vishwakarma et al., 2021
mLys/mAla	16	8	*P. aeruginosa*	Changes in bacterial surface properties that prevent the attachment Stimulating twitching motility	>1,250	Vishwakarma et al., 2021
mArg/mPhe	4	4	*P. aeruginosa*	Changes in bacterial surface properties that preventing the attachment Reducing swarming motility	197.5	Vishwakarma et al., 2021
mArg/mAla	4	4	*P. aeruginosa*	Changes in bacterial surface properties that prevent the attachment Stimulating twitching motility Reducing swarming motility	551.6	Vishwakarma et al., 2021
Nal-P-113	160	6.25	*P. gingivalis* W83	Downregulation of genes related to binding proteins, transport, motility and transposases		Wang et al., 2017a
DJK-5	16	1^a^	*P. aeruginosa*	Degradation of (p)ppGpp		De La Fuente-Núñez et al., 2015
	1.6	0.8^a^	*E. coli* 0157			
	8	4^a^	*A. baumannii*			
	3.2	1.6^a^	*K. pneumoniae*			
	3.2	0.8^a^	*S. enterica*			
DJK-6	16	0.5^a^	*P. aeruginosa*	Degradation of (p)ppGpp		De La Fuente-Núñez et al., 2015
	16	8^a^	*E. coli* 0157			
	8	2^a^	*A. baumannii*			
	4	2^a^	*K. pneumoniae*			
	4	1^a^	*S. enterica*			
Ac-Gup-Gup-Nap-Arg-NH2	>5 mM	0.08 (mM)^b^	*P. aeruginosa*	Sequestering c-di-GMP		Foletti et al., 2018
Ac-Nap-Gup-Gup-Nap-Arg-NH2	0.156 mM	0.02 (mM)^b^	*P. aeruginosa*	Sequestering c-di-GMP		Foletti et al., 2018

a Minimal biofilm inhibitory concentrations leading to 50% decrease in adherent biofilm growth.

b Minimal biofilm inhibitory concentrations that inhibited 50% of biofilm growth.

c HC_50_: peptidomimetic concentration that produces 50% of hemolysis.

Nal-P-113 (AKR-Nal-NalGYKRKF-Nal-NH2) is a salt-resistant peptidomimetic derived from the peptide P-113 *via* substitution of their histidine residues by β-naphthylalanine residues (Yu et al., 2011). Nal-P-113 appears to exert anti-biofilm activity against *Porphyromonas gingivalis* W83 biofilms through different mechanisms, dependent on peptide concentrations (Wang et al., 2017a). At high concentrations, Nal-P-113 exerts its anti-biofilm activity by killing the bacteria, whereas at sub-inhibitory concentrations (respective to planktonic growth), the exerted anti-biofilm activity appears to involve several molecular pathways, as suggested by gene expression data from microarray gene analysis between Nal-P-113-treated *P. gingivalis* W83 and non-treated bacteria (Wang et al., 2017a). In the Nal-P-113-treated bacteria, a significant downregulation was observed in genes that encode for cationic outer membrane proteins OmpH-1 and OmpH-2, which are recognized as virulence factors and have been detected in another study with upregulated expression on biofilms (Romero-Lastra et al., 2017). The *fimB* (PG2133) gene that encodes for FimB (Major Fimbrium Anchoring Subunit) was downregulated. FimB is involved in biofilm formation and adhesion onto host and bacterial cells (Uniprot, 2021). Then, sub-inhibitory concentrations of Nal-P-113 appear to induce surface changes in *P. gingivalis* that affect the establishment of biofilms. In line with this, scanning electron microscopy analysis of Nal-P-113-treated bacteria showed their inability to fuse together (Wang et al., 2017a). Furthermore, other sets of downregulated genes encode for ABC transporters. Previous experimental evidence suggests that ABC transporters could be involved in cell-to-surface and cell-to-cell interactions, the authors suggested that downregulation of these ABC transporter-encoding genes could also influence the inability of the Nal-P-113-treated bacteria to form biofilms (Kolenbrander et al., 1994; Matthysse et al., 1996; Hinsa et al., 2003). Genes PG1551 and PG1552, which encode for HmuY and HmuR proteins, were downregulated. HmuY and HmuR constitute a system that allows *P. gingivalis* uptake and use of heme (Olczak et al., 2008). Consequently, Nal-P-113-treated bacteria showed reduced usage of hemin in comparison with the control; since hemin availability appears to influence *P. gingivalis* capability to form biofilms (Dashper et al., 2012); the downregulated expression of PG1551 and PG1552 could be a pathway through which Nal-P-113 disturbs biofilm formation. It was observed that in Nal-P-113-treated bacteria, the transposase-encoding genes were downregulated; however, when bacteria are formerly treated with H_2_O_2_, the subsequent treatment with Nal-P-113 did not inhibit biofilm formation. Since earlier studies showed that, in *P. gingivalis*, H_2_O_2_ induces the expression of transposase genes, the results suggest that transposase genes’ downregulation by Nal-P-113 is important for its anti-biofilm activity (Diaz et al., 2006; McKenzie et al., 2015).

Another explored approach to hamper biofilms *via* peptidomimetics is by interfering with signaling molecules, especially alarmones, which are ribonucleotides or ribonucleotide derivatives involved in the bacterial adaptive response to environmental stresses. Pioneering work by De la Fuente-Núñez et al. (2015) showed the anti-biofilm potential of the peptidomimetics DJK-5 and DJK-6 (Table 2; De La Fuente-Núñez et al., 2015). Both DJK-5 and DJK-6 are D-enantiomers related to IDR-1018, a peptide that exerted an anti-biofilm activity *via* sequestering and further triggering the degradation of alarmones [(p)ppGpp] (De la Fuente-Núñez et al., 2014). Similarly to IDR-1018, DJK-5 and DJK-6 appear to exert their anti-biofilm activity by promoting the degradation of [(p)ppGpp]. DJK-5 and DJK-6 exerted potent *in vitro* broad-spectrum biofilm inhibitory activity against the wild-type and antibiotic-resistant strains of *P. aeruginosa*, *E. coli*, *A. baumannii*, *K. pneumoniae*, *S. enterica*, and *Burkholderia cenocepacia* (De La Fuente-Núñez et al., 2015). Moreover, the *in vivo* potential of DJK-5 and DJK-6 was demonstrated in biofilm-infection models, since both peptidomimetics protected *Caenorhabditis elegans* and *Galleria mellonella* against *P. aeruginosa* PAO1 lethal infections without exerting toxic effects. An important finding was that the combination of DJK-5 and DJK-6 with ceftazidime, imipenem, ciprofloxacin, or tobramycin inhibited biofilm formation. This allowed a reduction in the quantities of antibiotic needed, since the peptidomimetic-antibiotic interactions were often synergistic, near synergistic, or additive (De La Fuente-Núñez et al., 2015). Similarly, the peptidomimetic-antibiotic combination tended to be effective in eradicating mature biofilms (De La Fuente-Núñez et al., 2015). Further experimental evidence showed the potential of the combination of DJK-6 with β-lactam antibiotics to treat biofilms produced by carbapenemase-producing *K. pneumoniae* (KPC) clinical isolates (Ribeiro et al., 2015).

Studies focused on cutaneous abscess in mice shed additional evidence of the potential of DJK-5 as an anti-virulence drug either as monotherapy or in combination with antibiotics (Mansour et al., 2016; Pletzer et al., 2017, 2018). A stringent response of bacterial pathogens like MRSA and *P. aeruginosa* appears to be linked to abscess pathology. In line with this, in CA-MRSA USA300 and *P. aeruginosa* LESB58-infected mice treated with DJK-5, there was a reduction in the pathological manifestations of abscess (Mansour et al., 2016). It was also observed that DJK-5 treatment repressed the production of phenol-soluble modulin (PSM) toxins by *S. aureus*, which could contribute to virulence attenuation since PMS toxins mediate tissue damage and immune response evasion (Mansour et al., 2016). A later study demonstrated the effectiveness of DJK-5 in treating cutaneous abscesses caused by *P. aeruginosa* strains. DJK-5-treated animals showed reduced dermonecrosis and bacterial counts at the abscess site (Pletzer et al., 2017). A remarkable observation was that DJK-5 suppressed *rpo*Z-*spoT* operon *in vivo* expression. Since *rpo*Z (ω subunit of the RNA polymerase) could influence *relA* (encode for RelA, the enzyme that synthesizes pppGpp) expression, it was hypothesized that in addition to binding and triggering ppGpp degradation, DJK-5 could also disrupt ppGpp levels through the downregulation of *relA* expression (Pletzer et al., 2017). Furthermore, based on a cutaneous mouse infection model, more evidence of the anti-virulence potential of DJK-5 was added, since animals treated with DJK-5 tended to have reduced bacterial load in the infection site. Combinatory DJK-5 therapy with ciprofloxacin, meropenem, erythromycin, gentamicin, and vancomycin diminished abscess sizes and/or reduced bacterial load in murine cutaneous abscesses caused by *E. coli* and ESKAPE pathogens (Pletzer et al., 2018). It was proposed that DJK-5 could synergize with antibiotics *via* disrupting the stringent-stress response and breaking the permeability barrier, which could facilitate antibiotic penetration (Pletzer et al., 2018).

Peptidomimetics that target the (p)ppGpp-mediated stringent response could be promising anti-virulence drugs to cope with biofilm-associated infections. The stringent response appears to be involved in biofilm development, a rise in persistence, abscess formation, and host-environment adaptation (Alford et al., 2019; Kundra et al., 2020). Although biofilms and abscesses are physiologically different, both are high-density bacterial infections that tend to be recalcitrant to antibiotic therapy (Mansour et al., 2016; Pletzer et al., 2018). Moreover, the (p)ppGpp-mediated stringent response is broadly distributed in bacteria and absent in mammals, which made it an attractive target for the development of drugs with broad-spectrum activity and low toxicity (De la Fuente-Núñez et al., 2014; Pletzer and Hancock, 2016).

In addition to (p)ppGpp, cyclic di-guanosine (c-di-GMP) is another second-messenger nucleotide that have been targeted by peptidomimetics (Foletti et al., 2018; Hee et al., 2020). Recently, functionalized proline-rich peptides with the capacity to bind selectively to c-di-GMP showed anti-biofilm activity against *P. aeruginosa* biofilms (Foletti et al., 2018). The peptides that bonded to c-di-GMP with higher affinity and selectivity contained three cationic residues of guanidinoproline (Gup) and/or arginine (Arg) and the aromatic group naphthyl (Nap) (Figure 3). This arrangement of aromatic and cationic residues is essential for the binding to c-di-GMP through π-π, electrostatic and H-bonding interactions. The peptides were assayed to evaluate their biofilm inhibitory capacity, and Ac-Gup-Gup-Nap-Arg-NH2 and Ac-Nap-Gup-Gup-Nap-Arg-NH2 were the most active peptides (Table 2; Foletti et al., 2018). Another study reported a shortened and modified c-di-GMP-sequestering peptide (CSP4) derived from CleD (a CheY-like c-di-GMP effector protein) (Hee et al., 2020). CSP4 is 19 amino acids residues in length, with its N-terminal acetylated and its C-terminal amidated, which bind to c-di-GMP specifically and with high affinity. Although CSP4 binds to c-di-GMP, when it was expressed in *P. aeruginosa* either alone or fused with Venus fluorescent protein, no effect was observed on biofilm formation. However, the expression of another c-di-GMP-sequestering peptide variant (CSP2) as a fused protein with maltose-binding protein (MBP) and hexahistidine (H6) (H6-MBP-CSP2) inhibited the biofilm formation (Hee et al., 2020). Further studies should be addressed to verify if c-di-GMP-sequestering peptides could penetrate the biofilm structure and sequester c-di-GMP.

Similarly to (p)ppGpp, peptidomimetics that target c-di-GMP, lowering its levels, could be a promising anti-virulence strategy, since c-di-GMP plays a critical role in the virulence of several medically important pathogens (Hall and Lee, 2018; Valentini and Filloux, 2019). However, it is important to consider that the effect of c-di-GMP levels on virulence could be specific to pathogens and type of infection (Hall and Lee, 2018). An additional element that made c-di-GMP an interesting target is that it is not biosynthesized by humans (Andersen et al., 2021).

## Conclusion

Currently, antimicrobial resistance is a major concern for human health. The emergence and spread of multidrug resistant pathogens are forcing the search for non-conventional therapeutic strategies. In this regard, anti-virulence therapy is one of the non-conventional approaches that is being explored, and it aims to disrupt pathogen virulence but tries to exert marginal selective pressure. As a nascent strategy, it is necessary to discover, design, and develop anti-virulence compounds. Natural peptide-based drugs act as anti-virulence compounds but present some pharmacological shortcomings. Instead, peptidomimetics have improved pharmacologic properties, so they could be a valuable source of anti-virulence drugs. Although peptidomimetics have been widely explored as antimicrobials, experimental evidence supports their potential as anti-virulence drugs. Among the virulence factors and virulence-associated processes targeted by peptidomimetics are bacterial secretion systems, quorum-sensing systems, and biofilms. Bacterial secretion systems are an attractive target for the development of anti-virulence drugs, due to their link with virulence-associated processes, such as host invasion and immune system evasion. Moreover, their wide distribution in bacterial pathogens makes it possible to develop pathogen-specific anti-virulence drugs. Due to their multiprotein composition, protein–protein interactions are a particularly important process in secretion system assembly and function; consequently, protein–protein interactions could be a target to be exploited for the development of novel peptidomimetics with anti-virulence activity. Another key target for the development of peptidomimetic-based anti-virulence drugs is quorum-sensing systems, since they regulate the expression of several virulence factors. Several peptidomimetics have been designed to target quorum-sensing systems dependent on autoinducing peptides. Although peptidomimetics have been mainly involved in SAR studies, these promising molecules could be interesting for pharmacological development due to their advantages. Interestingly, in some cases, peptidomimetics keep their specificity on bacterial receptors and enhance agonistic activity, but most importantly, they can antagonize several receptors simultaneously. In SAR studies, the quorum-sensing system most targeted by peptidomimetics has been the Agr system from *Staphylococci* spp., whereas the RRNPP pathway has been poorly explored. Consequently, few peptidomimetics have been designed to target the RRNPP system, which reveals the lack of knowledge about this system’s action mechanism. However, for the peptide-based QS pathway, several peptidomimetic candidates appear to be promising for subsequent studies and biotechnological development.

Peptidomimetics are also an emerging approach to tackle biofilms, showing promising results. However, in the context of anti-virulence therapy, it is necessary to understand how the anti-biofilm activity of peptidomimetics could influence the production of virulence factors, since virulence factor production may be affected by the type of bacterial growth (Chua et al., 2014; Guilhen et al., 2016; Quinn et al., 2021). In this context, for example, the disintegration of biofilms could yield biofilm-dispersed cells with virulence factor profiles different to biofilm and planktonic growth states (Chua et al., 2014; Guilhen et al., 2016, 2017). Biofilm-dispersed bacteria could have a superior capacity for colonization and evasion of host immune response, thus increasing their virulence (Chua et al., 2014; Guilhen et al., 2017, 2019).

Overall, peptidomimetics appear to be promising anti-virulence drugs. However, it is necessary to get inside their anti-virulence mechanisms and it is important to develop more studies focused on assessing their efficacy *in vivo*.

## Author Contributions

HD performed figure design. All authors listed have made a substantial, direct, and intellectual contribution to the work and approved it for publication.

## Funding

This work was supported by the CAPES, CNPq, FUNDECT, and FAPDF.

## Conflict of Interest

The authors declare that the research was conducted in the absence of any commercial or financial relationships that could be construed as a potential conflict of interest.

## Publisher’s Note

All claims expressed in this article are solely those of the authors and do not necessarily represent those of their affiliated organizations, or those of the publisher, the editors and the reviewers. Any product that may be evaluated in this article, or claim that may be made by its manufacturer, is not guaranteed or endorsed by the publisher.
